# Simultaneously performed combined 24-2 and 10-2 visual field tests in glaucoma

**DOI:** 10.1038/s41598-020-80318-w

**Published:** 2021-01-13

**Authors:** Kyoung In Jung, Hee Kyung Ryu, Ki Hoon Hong, Yong Chan Kim, Chan Kee Park

**Affiliations:** 1 College of Medicine, The Catholic University of Korea, Seoul, Korea; 2 Department of Ophthalmology, Seoul St. Mary’s Hospital, College of Medicine, The Catholic University of Korea, 222 Banpo-daero, Seocho-ku, Seoul, 137-701 Korea; 3 Department of Ophthalmology, Eunpyung St. Mary’s Hospital, Seoul, Korea; 4 Department of Ophthalmology, Incheon St. Mary’s Hospital, Incheon, Korea

**Keywords:** Optic nerve diseases, Eye diseases, Glaucoma

## Abstract

Using either 24-2 or 10-2 visual field (VF) testing only is not enough to cover all the various types of glaucomatous VF defects. We investigated the performance of the combined 24-2 and 10-2 perimetry when conducted together and separately using the structure–function relationship. A total of 30 glaucoma patients with isolated peripheral nasal step, 37 patients with isolated paracentral scotoma, and 38 patients with both paracentral and nasal scotoma were included. To create the combined Humphrey VF test, a custom test pattern was established using the built-in custom point options, an example of the X, Y coordinate system. In glaucoma patients with peripheral nasal step, the superotemporal topographic structure–function relationship with peripapillary retinal nerve fiber layer (RNFL) thickness was superior in relation to the combined or 24-2 perimetry relative to the 10-2 perimetry (both P < 0.05). The combined VF test showed more favorable inferotemporal or inferonasal structure–function correlation with the corresponding ganglion cell–inner plexiform layer (GCIPL) thickness when compared with results gleaned using the 24-2 VF test (P < 0.05). Simultaneously performed 24-2 and 10-2 VF tests demonstrated a superior topographic structure–function relationship when compared with them separately performed in some sectors.

## Introduction

In patients with glaucoma, 24-2 standard automated perimetry (SAP) has been considered as the most commonly applied perimetry to examine the visual function. However, accumulating evidence has reported that the 24-2 SAP can underestimate glaucomatous damage in the macular area^1,2^. The reason for this could be because six degrees of spacing of test points in the 24-2 visual field (VF) test is not enough to cover the more densely concentrated retinal ganglion cells in the paracentral retina.

Many reports have highlighted the favorable performance of 10-2 SAP with more test points of two degrees of spacing in the paracentral region in the early detection of glaucomatous macular damage^2,3^. De Moraes et al. found that 61.5% of results categorized as normal based on 24-2 VF testing were defined as abnormal on 10-2 VF testing among early glaucoma patients^3^. On the other hand, peripheral nasal step, commonly occurring in patients with early glaucoma, could not be detected with 10-2 SAP. Elsewhere, Traynis et al. reported that approximately 20% of the 24-2 SAP results categorized as abnormal were determined to be normal on the 10-2 VF test^2^. Therefore, using either 24-2 or 10-2 VF testing only is not enough to cover all the various types of glaucomatous VF defects.

In clinical practice, implementing both 24-2 and 10-2 SAP requires more time and effort on the part of patients. Physicians should strive to record more VF images to detect and judge glaucomatous functional damage. The combination of 24-2 and 10-2 VF testing into a single VF test might be helpful to avoid not only missing early glaucomatous damage but also wasting time.

Enrlich et al. demonstrated that adding four or 16 points from the 10-2 SAP to the 24-2 SAP enhanced its capacity to detect macular damage in glaucoma patients^4^. In their study, however, a particular subset of points from the 10-2 VF tests was added retrospectively to the 24-2 VF test after both were separately performed^4^.

The 24-2C grid, a new test pattern available on the Humphrey SITA-Faster algorithm, adds 10 selected points from the 10-2 VF grids that include regions found to be vulnerable to glaucomatous damage. The 24-2C test tended to identify more clusters of central VF defects than the 24-2 test, but the difference was not statistically significant^5^. Checking the results when all points from the 10-2 VF are added to the 10 degrees of visual points of the 24-2 VF is warranted.

In this study, we created a customized combined VF test pattern using the built-in custom point options with the X, Y coordinate system in the Humphrey VF test. We then compared the performance of combined 24-2 and 10-2 perimetries conducted simultaneously and separately, respectively, among glaucoma patients with different types of scotoma using a structure–function relationship.

## Methods

### Subjects

This cross-sectional study was permitted by the institutional review board of the Catholic University of Korea, Seoul, Korea and was conducted in compliance with the tenets of the Declaration of Helsinki. Glaucoma patients who met the inclusion criteria were included at the Glaucoma Clinic of Seoul St. Mary’s Hospital between May 2016 and August 2018. Informed consent was obtained from all subjects. Eyes displaying glaucomatous optic discs such as rim thinning, notching, or retinal nerve fiber layer (RNFL) defects with corresponding glaucomatous VF loss were included in this study. Patients were excluded if they showed best-corrected visual acuity < 20/30, axial lengths of longer than 28 mm, or a history of brain disease or trauma. Eyes with uveitis or retinal disease that could influence visual acuity or visual field were also excluded.

### Measurements

All subjects performed best-corrected visual acuity, slit-lamp biomicroscopy, tonometry, axial length determination (IOLMaster; Carl Zeiss Meditec, Dublin, California, USA), and fundus photography.

#### Optical coherence tomography

Using the Cirrus HD-OCT version 6.0 system (Carl Zeiss Meditec, Jena, Germany). The optic disc cube scan mode was selected. Cirrus-OCT system identifies the middle of the disc and then depicts a circumpapillary circle from the cube dataset for RNFL thickness analysis. The average or quadrant RNFL thickness was analyzed. Separately, applying a macular cube scan approach, the ganglion cell-inner plexiform layer (GCIPL) thickness was analyzed. Ganglion-cell analysis was adopted to measure the average, minimum, and sectoral (i.e., superior, superotemporal, superonasal, inferior, inferotemporal, and inferonasal) GCIPL thickness parameters. Poor-quality images with a signal strength < 6 were excluded.

#### VF testing

All subjects carried out 24-2, 10-2, and combined SAP using a Humphrey field analyzer (Carl Zeiss Meditec, Jena, Germany). Reliable VF examinations were considered as those having less than 15% fixation losses, false positives, or false negatives. A glaucomatous VF loss was defined as having ≥ 3 points with a *P* < 0.05 of being normal, one of which displayed a *P* < 0.01 for the pattern deviation plot of 24-2 SAP.

Mean sensitivity (MS) was measured on threshold printouts. VF sensitivity was analyzed using the logarithmic dB scale. Overall MS was calculated as the average of VF sensitivities in the 52 points from the 24-2 VF, 68 points in the 10-2 VF, and 108 points in the combined VF, respectively.

The Swedish interactive threshold algorithm (SITA) standard program with Goldmann size III targets was adopted for 24-2 and 10-2 SAP. The mean deviation (MD) and pattern standard deviation (PSD) were analyzed for 24-2 and 10-2 SAP.

### Creation of the combined 24-2 and 10-2 VF test

**Figure 1:**
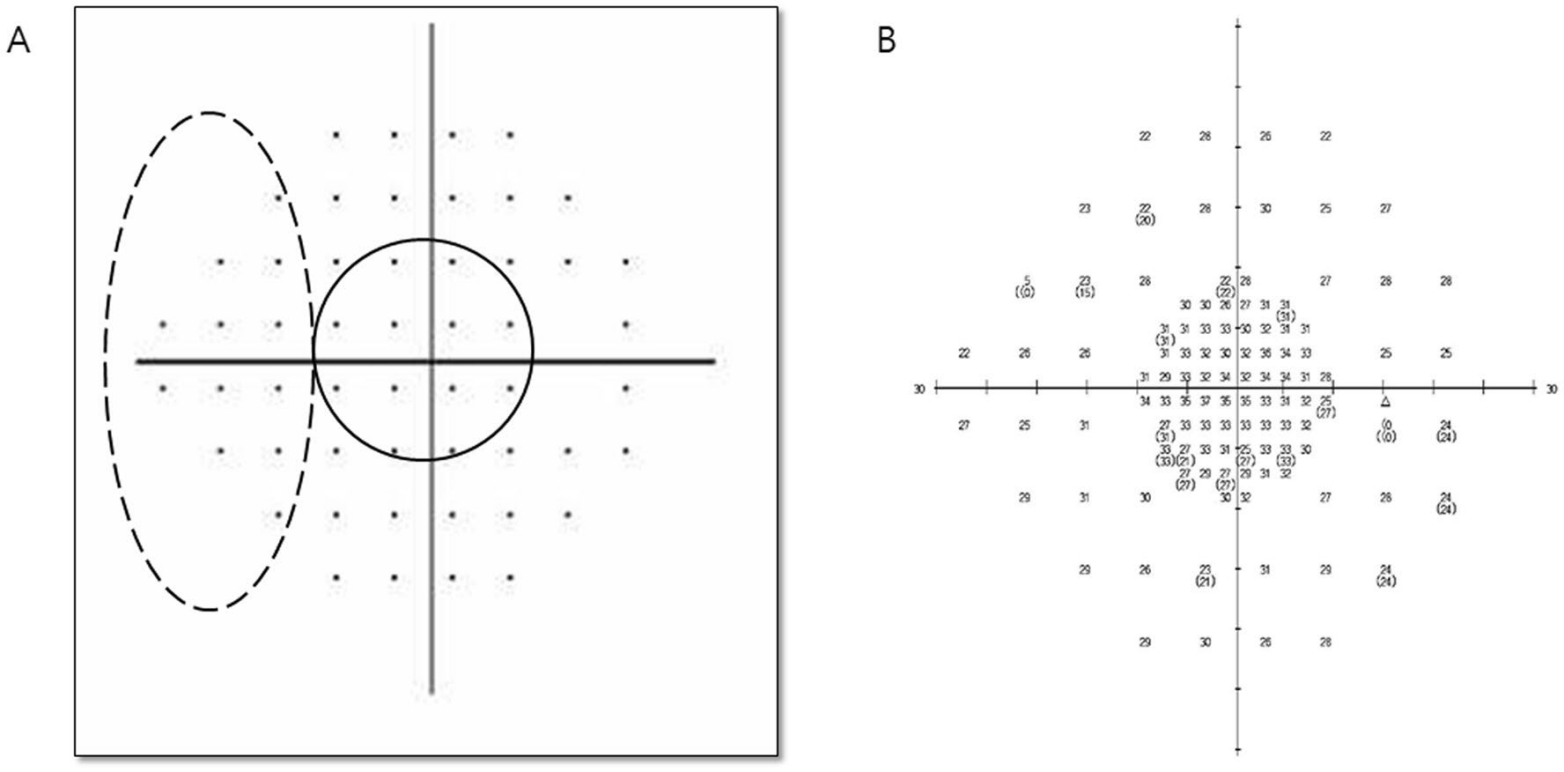
(**A**) Paracentral scotoma (12 points within the dashed line) and peripheral nasal step (12 points within the dotted line) shown in the PSD plot of the 24-2 VF test (**B**) Representative simultaneously performed combined 24-2 and 10-2 VF test.

Using the built-in custom test options, the custom test pattern was made with the X, Y coordinate system in the Humphrey VF test. A total of 110 coordinates were input to produce the combined 24-2 and 10-2 grid patterns, the example of which is shown in Fig. 1. An analysis was performed for 108 points, excepting two blind spot points. In the combined VF test, the full threshold strategy was applied because of the unavailability of the SITA strategy for the customized VF patterns. The MD or PSD was not provided for the customized combined VF test. Goldmann size III targets were used also to the combined VF test.

The combined 24-2 and 10-2 VF analyzed in this study consisted of 108 points, excluding the two blind spots. The 24-2 VF test includes 52 points excepting two blind spots and the 10-2 VF test includes 68 points. The one hundred eight test points of the combined VF test numbered 12 points less than the sum (120 points) of points of the separately performed 24-2 and 10-2 VF tests.

### VF criteria for paracentral scotoma and peripheral nasal step

The isolated paracentral scotoma and the isolated peripheral nasal step groups were established by a glaucoma specialist (K. I. J.) on the basis of pattern deviation probability plots acquired from the 24-2 perimetry. Paracentral scotoma subjects displayed isolated glaucomatous VF loss within 12 points of a central 10° of fixation in 1 hemifield (Fig. 1), while peripheral nasal step subjects presented isolated glaucomatous VF loss within the nasal periphery outside 10° of fixation in 1 hemifield. Glaucoma patients with both paracentral scotoma and peripheral nasal scotoma were assigned to the paracentral scotoma and peripheral nasal scotoma group.

### Structure–function relationship

**Figure 2:**
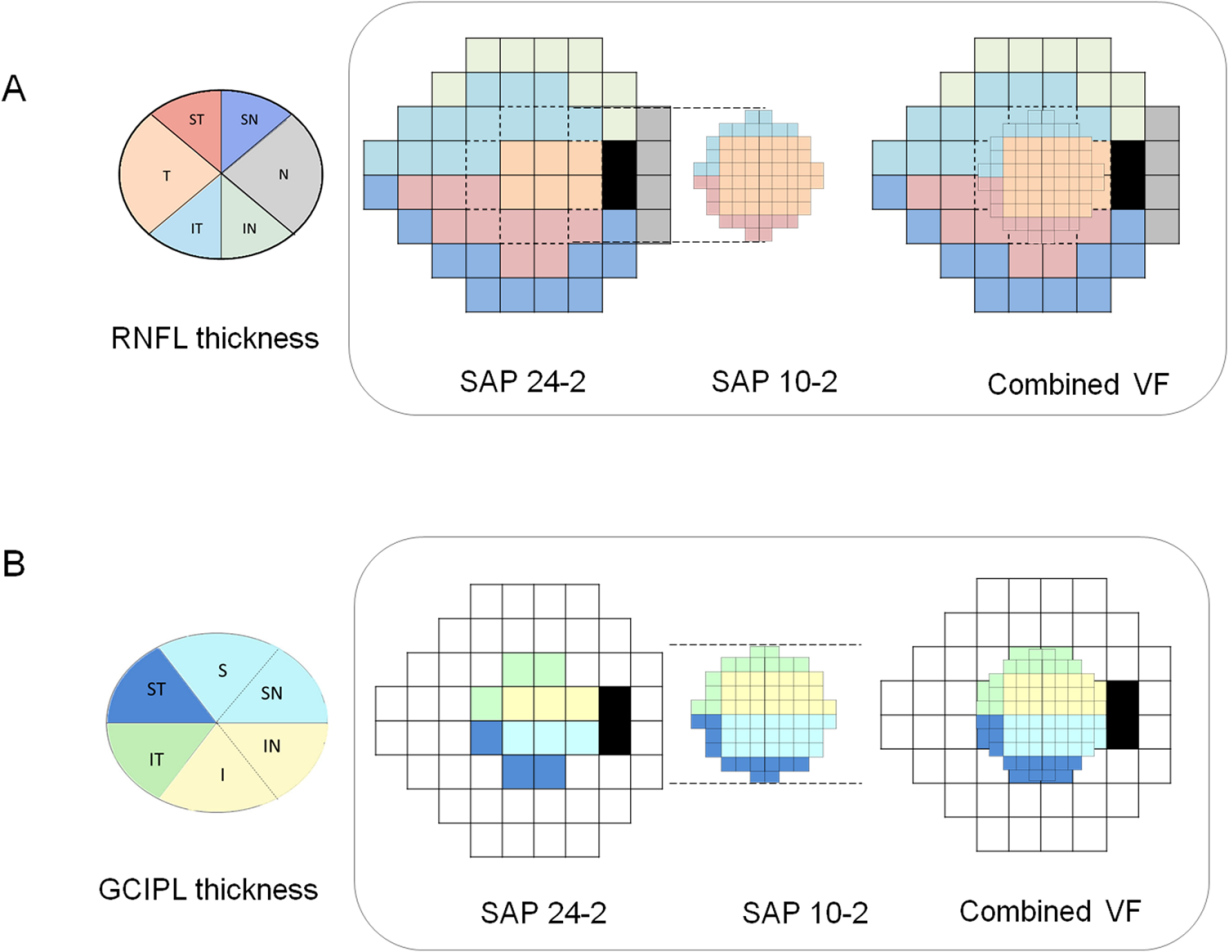
Structure–function correspondence map according to Garway-Heath et al^7^. (**A**) Topographic RNFL thickness and the corresponding VF sectors in 24-2 or 10-2 SAP and the combined VF test (**B**) Topographic GCIPL thickness and the corresponding 24-2 or 10-2 SAP or the combined VF test. The figure was prepared using the Excel and PowerPoint program in Microsoft Office 365 (https://www.office.com/, freely available for teachers at qualified academic institutions). I = inferior; IN = inferonasal; IT = inferotemporal; N = nasal; S = superior; SN = superonasal; ST = superotemporal; T = temporal.

Average (360° measure), superonasal (91–135°), nasal (136–225°), inferonasal (226–270°), inferotemporal (271–315°), temporal (316–45°), and superotemporal (46–90°) RNFL thickness was analyzed in this study. The RNFL thickness of each sector was calculated by incorporating the clock-hour RNFL thickness from the Cirrus HD-OCT system^6^. We adjusted these sectors in compliance with the structure–function correspondence map developed by Garway-Heath et al. (Fig. 2)^7^. Sixty-eight VF test points on 10-2 SAP, 52 VF test points except for two blind spots on 24-2 SAP, and 108 points on combined SAP were allocated to superotemporal, inferotemporal, superonasal, and inferonasal sectors by applying the Garway-Heath et al. map designed for 24-2 SAP (Fig. 2)^7^.

Twelve VF test points from 24-2 SAP and 68 points from 10-2 SAP or combined SAP were allocated to superotemporal, inferotemporal, superonasal, and inferonasal sectors by adjusting the Garway-Heath map designed for 24-2 VF tests (Fig. 2). For the superotemporal and inferotemporal topographical structure–function relationship, superotemporal and inferotemporal GCIPL thicknesses were adopted as it is. The sum of the superior and superonasal GCIPL thicknesses was used for the superonasal sector, and the sum of the inferior and inferonasal GCIPL thicknesses was used for inferonasal sector GCIPL thickness.

### Statistical method

Statistical analysis was completed using the Statistical Package for the Social Sciences version 22.0 software program (IBM Corp., Armonk, NY, USA). Differences between two groups were estimated using the Student’s *t*-test or chi-squared test. Differences among the three groups were analyzed by one-way analysis of variance. Correlations between the parameters were evaluated according to Pearson’s correlation coefficient. To compare the correlation between VF tests, a Hotelling–Williams test was adopted. To evaluate point-wise correlations between VF tests, the intraclass correlation coefficient (ICC) was used, with scores of 0.75 or more, 0.40 to 0.75, and 0.40 or less points considered to be excellent, moderate, and poor, respectively^8^. A P-value of less than 0.05 indicated statistical significance.

## Results

A total of 30 glaucoma patients with peripheral nasal step, 37 patients with paracentral scotoma, and 38 patients with both paracentral scotoma and peripheral nasal scotoma were included in this study after the exclusion of three patients presenting low reliability of the combined 24-2 and 10-2 VF test. The demographics exhibited no significant differences between the three groups (all P > 0.05) (Table 1). The average RNFL thickness was less in the paracentral scotoma and peripheral nasal scotoma group than in the paracentral scotoma or peripheral nasal step groups (P < 0.001). Separately, the average GCIPL thickness was less in the paracentral scotoma or the paracentral scotoma and peripheral nasal scotoma group than in the peripheral nasal step group (P < 0.001).

**Table 1:** Characteristics of glaucoma patients.

Parameter	Isolated peripheral nasal step (n = 30)	Isolated paracentral scotoma (n = 37)	Paracentral & nasal scotoma (n = 38)	*P* value	Post hoc* analysis*
Age (years)	53.4 ± 14.2	54.3 ± 11.8	50.0 ± 12.0	0.310	
Male/female	18/12	15/22	15/23	0.177	
Central corneal thickness (µm)	532.5 ± 43.0	521.5 ± 39.6	528.9 ± 40.7	0.546	
Spherical equivalent (diopter)	− 3.1 ± 3.0	− 2.4 ± 2.8	− 3.2 ± 2.9	0.413	
Axial length (mm)	25.1 ± 1.6	24.5 ± 1.6	24.9 ± 1.3	0.395	
Average RNFL thickness (µm)	76.2 ± 8.8	76.2 ± 9.6	67.4 ± 9.4	** < 0.001**	Group 3 < Group 1, 2
Average GCIPL thickness (µm)	73.6 ± 7.0	67.1 ± 6.9	66.1 ± 7.6	** < 0.001**	Group 2, 3 < Group 1

Statistically significant values (*P* < 0.05) are shown in bold.

GCIPL, ganglion cell-inner plexiform layer; RNFL, retinal nerve fiber layer.

Continuous variables are expressed as n (percentage), mean ± standard deviation, or percentage.

Post hoc analysis by Turkey’s-b, Group 1: Isolated peripheral nasal step, Group 2: Isolated paracentral scotoma, Group 3: Paracentral & nasal scotoma.

The MD on 24-2 SAP was lower than that on 10-2 SAP in the peripheral nasal step group but was higher than that on 10-2 SAP in the paracentral scotoma group (both P < 0.001) (Table 2). The PSD on 24-2 SAP was higher than that on 10-2 SAP in the peripheral nasal step group but was lower than that on 10-2 SAP in the paracentral scotoma group (P = 0.002 and P < 0.001, respectively). MD or PSD presented no significant difference between the 24-2 SAP and 10-2 SAP in the paracentral and nasal scotoma group (P = 0.240 and P = 0.380, respectively).

**Table 2:** Mean deviation and pattern standard deviation of standard automated perimetry in glaucoma patients.

Parameter	Type of perimetry	Isolated peripheral nasal step (n = 30)	Isolated paracentral scotoma (n = 37)	Paracentral & nasal scotoma (n = 38)
MD (dB)	SAP 24-2	− 2.8 ± 2.8	− 2.3 ± 1.7	− 8.0 ± 5.3
	SAP 10-2	− 0.6 ± 1.8	− 4.6 ± 3.1	− 9.2 ± 5.7
	*P* value	** < 0.001**	** < 0.001**	0.240
PSD (dB)	SAP 24-2	4.8 ± 3.2	4.4 ± 2.2	9.9 ± 3.6
	SAP 10-2	2.4 ± 2.4	6.6 ± 4.1	10.5 ± 4.4
	*P* value	**0.002**	** < 0.001**	0.383

Statistically significant values (*P* < 0.05) are shown in bold.

MD, mean deviation; PSD, pattern standard deviation; SAP, standard automated perimetry.

*Differences between the SAP 24-2, SAP 10-2 were compared by paired t-test.

Mean test duration of the combined VF was 23.6 ± 3.5 min, which was longer than that for the 24-2 VF (5.7 ± 0.9 min) or the 10-2 VF (6.3 ± 1.2 min) (both P < 0.001).

**Figure 3:**
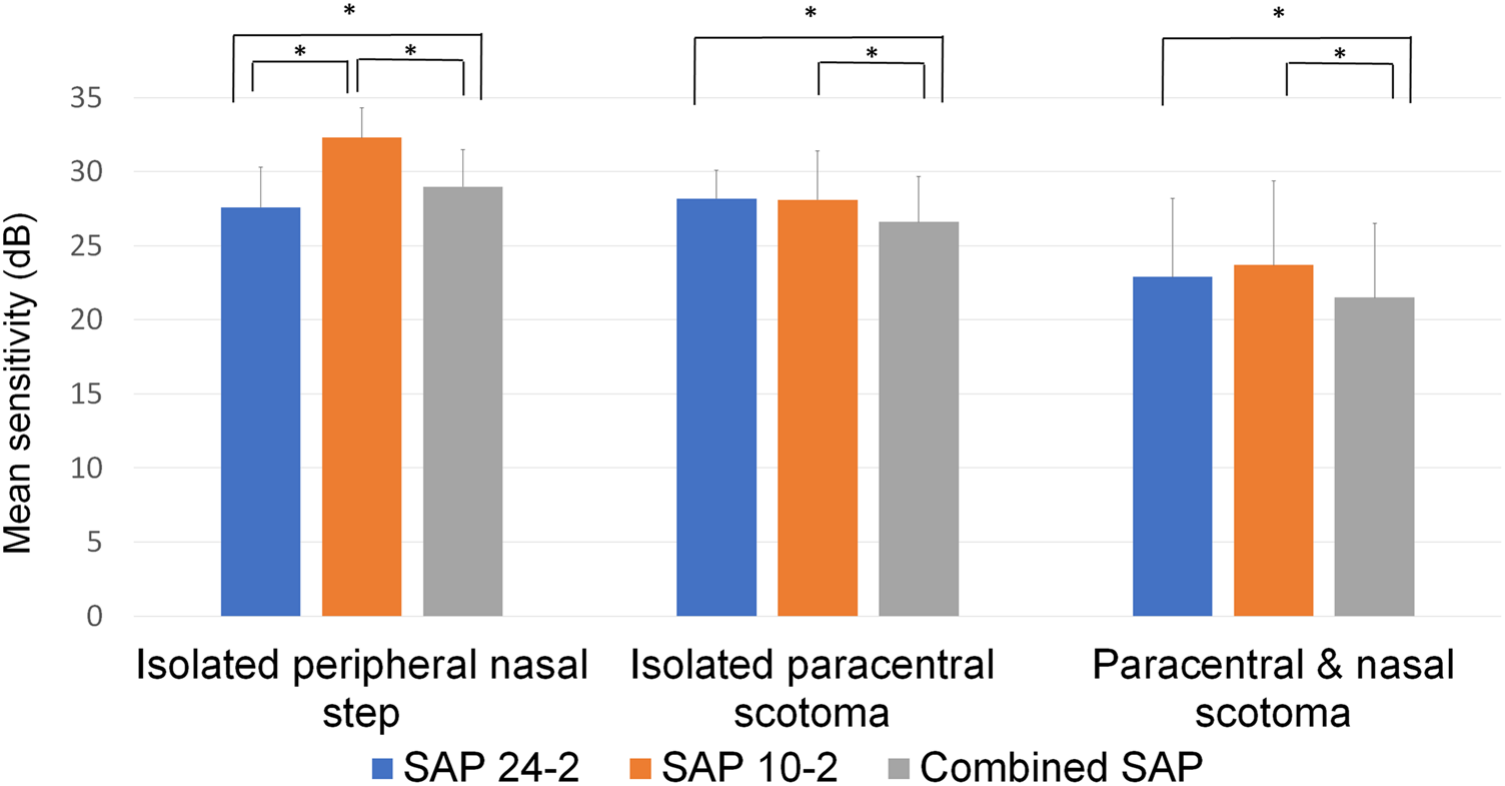
Global MS of the VF test. In the peripheral nasal step group, the MS was decreased in the 24-2 VF test and the combined VF test when compared with in the 10-2 VF test (both P < 0.001). In the isolated paracentral scotoma group and the paracentral and nasal scotoma group, the MS was the smallest in correlation with the combined VF test among the three VF tests (all P < 0.005).

In the peripheral nasal step group, the MS was less following the 24-2 VF test and the combined VF test relative to after 10-2 VF test (both P < 0.001) (Fig. 3). The combined SAP displayed the least MS among three VF tests in the isolated paracentral scotoma group and the paracentral and nasal scotoma group (all P < 0.005).

In the topographic structure–function relationship with peripapillary RNFL thickness, all three VF tests showed positive correlations in the superotemoral and inferotemporal sectors in the isolated peripheral nasal step group (all P < 0.05) (Table 3). Meanwhile, in the superotemporal sector, the correlation was higher with the combined SAP or 24-2 SAP than with 10-2 SAP (P = 0.041 and P = 0.018, respectively). In the isolated paracentral scotoma and the paracentral and nasal scotoma group, there was no significant difference in the structure–function relationship between the VF tests (P > 0.05).

**Table 3:** Topographic structure–function relationship between regional visual field sensitivity measured with standard automated perimetry 24-2 or standard automated perimetry 10-2 or combined SAP and the corresponding peripapillary retinal nerve fiber layer (RNFL) thickness.

Visual field tests	SD-OCT RNFL thickness	Isolated peripheral nasal step (n = 30)		Isolated paracentral scotoma (n = 37)		Paracentral & nasal scotoma (n = 38)	
		r	*P* value	r	*P* value	r	*P* value
SAP 24-2	ST	0.682*	**< 0.001**	0.296	0.071	**0.545**	** < 0.001**
	IT	**0.460**	**0.010**	**0.405**	**0.012**	**0.385**	**0.017**
	T	0.121	0.523	− 0.158	0.343	**0.421**	**0.009**
	SN	0.210	0.265	0.175	0.294	**0.537**	**0.001**
	IN	0.228	0.225	0.115	0.493	**0.340**	**0.037**
	N	− 0.034	0.860	− 0.011	0.946	− 0.151	0.364
SAP 10-2	ST	**0.374**	**0.042**	**0.379**	**0.019**	**0.685**	** < 0.001**
	IT	**0.385**	**0.036**	**0.342**	**0.047**	**0.359**	**0.027**
	T	− 0.114	0.550	− 0.181	0.277	**0.342**	**0.036**
Combined SAP	ST	**0.606**	** < 0.001**	0.304	0.067	**0.586**	** < 0.001**
	IT	**0.397**	**0.030**	0.259	0.121	**0.457**	**0.004**
	T	0.019	0.920	− 0.035	0.838	**0.403**	**0.012**
	SN	0.206	0.275	0.126	0.458	**0.538**	**0.001**
	IN	− 0.030	0.876	0.061	0.720	0.119	0.477
	N	0.007	0.972	− 0.124	0.463	− 0.225	0.174

Statistically significant values (*P* < 0.05) are shown in bold.

RNFL, retinal nerve fiber layer; SAP, standard automated perimetry; SD-OCT, spectral domain optical coherence tomography.

r = Pearson’s correlation coefficient.

*Statistically significant difference with P < 0.05 between SAP 24-2 and SAP 10-2.

^†^Statistically significant difference with P < 0.05 between SAP 24-2 and combined SAP.

^‡^Statistically significant difference with P < 0.05 between SAP 10-2 and combined SAP.

In the topographic structure–function relationship with GCIPL thickness, all VF tests demonstrated significant positive correlations in the paracentral and nasal scotoma group (all P < 0.005) (Table 4). The combined SAP revealed higher correlations at the inferotemporal and inferonasal sectors when compared with the 24-2 SAP in the paracentral and nasal scotoma group (P = 0.002 and 0.021, respectively). In the peripheral nasal step group and paracentral scotoma group, comparisons between any VF tests did not display a significant difference in the structure–function relationship with GCIPL thickness (all P > 0.005).

**Table 4:** Topographic structure–function relationship between regional visual field sensitivity measured with standard automated perimetry 24-2 or standard automated perimetry 10-2 or combined SAP and the corresponding ganglion cell-inner plexiform layer(GCIPL) thickness in glaucoma patients.

SD-OCT GCIPL Sector	Isolated peripheral nasal step (n = 30)		Isolated paracentral scotoma (n = 37)		Paracentral & nasal scotoma (n = 38)	
	r	*P* value	r	*P* value	r	*P* value
**SAP 24-2**						
ST	0.221	0.241	0.303	0.068	**0.627**	** < 0.001**
IT	0.235	0.210	0.397	0.074	**0.464**	**0.003**
SN	0.183	0.333	0.308	0.064	**0.661**	** < 0.001**
IN	0.284	0.128	**0.514**	**0.001**	**0.524**	**0.001**
**SAP 10-2**						
ST	0.124	0.504	**0.387**	**0.018**	**0.651**	** < 0.001**
IT	**0.533**	**0.002**	**0.435**	**0.007**	**0.555**	** < 0.001**
SN	0.176	0.353	0.190	0.261	**0.678**	** < 0.001**
IN	0.286	0.126	**0.373**	**0.023**	**0.629**	** < 0.001**
**Combined SAP**						
ST	0.203	0.281	**0.393**	**0.016**	**0.687**	** < 0.001**
IT	**0.515**	**0.004**	**0.543**	**0.001**	**0.676**†	** < 0.001**
SN	– 0.043	0.822	0.216	0.199	**0.677**	** < 0.001**
IN	0.283	0.130	**0.358**	**0.030**	**0.707**†	** < 0.001**

Statistically significant values (*P* < 0.05) are shown in bold.

GCIPL, Ganglion cell-inner plexiform layer; SAP, standard automated perimetry; SD-OCT, spectral domain optical coherence tomography.

r = Pearson’s correlation coefficient.

*Statistically significant difference with P < 0.05 between SAP 24-2 and SAP 10-2.

^†^Statistically significant difference with P < 0.05 between SAP 24-2 and combined SAP.

^‡^Statistically significant difference with P < 0.05 between SAP 10-2 and combined SAP.

**Figure 4:**
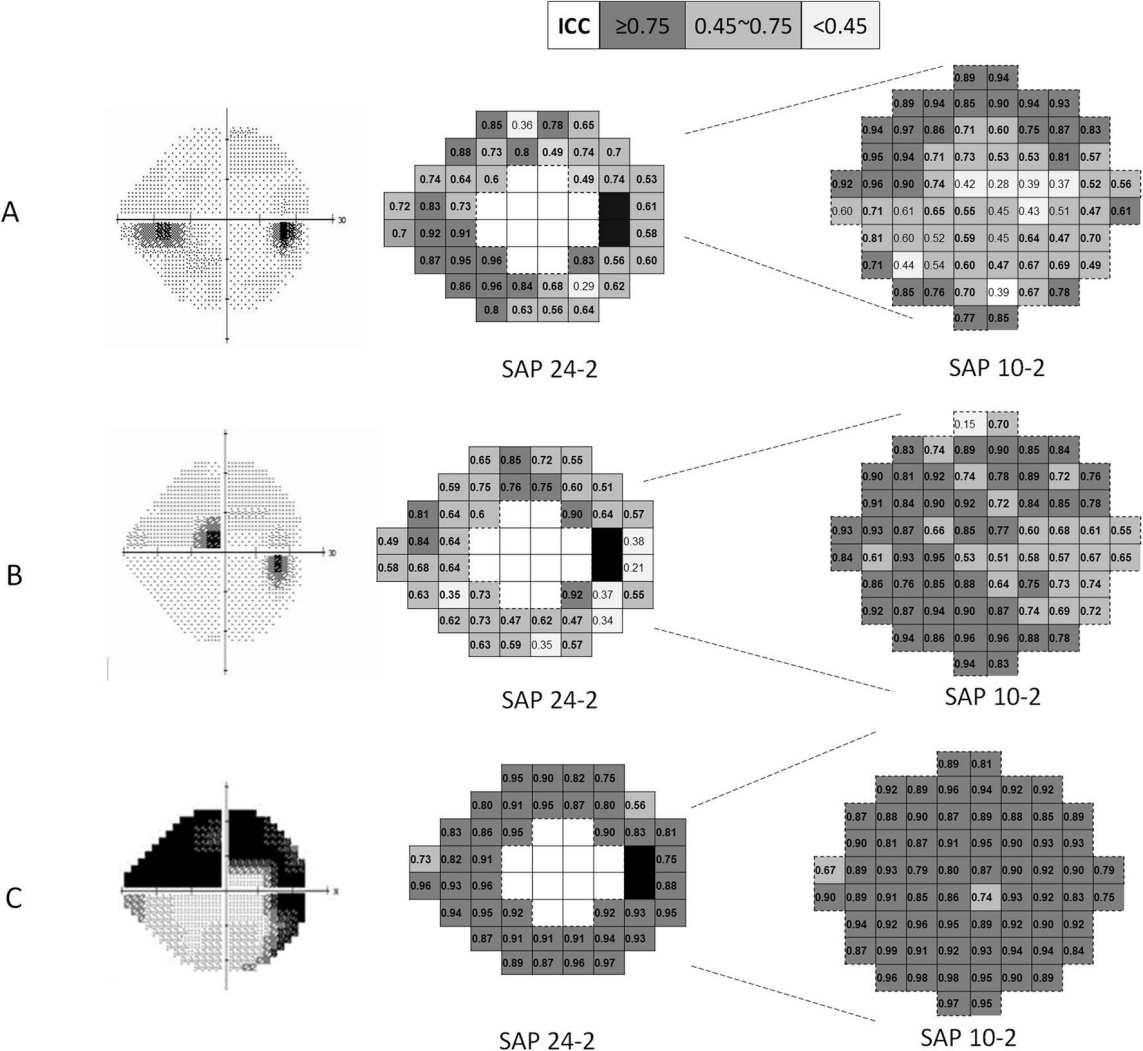
Point-wise ICC between the combined VF test and 24-2 VF test or 10-2 VF test. (**A**) In the peripheral nasal step group, the agreement between the 24-2 VF test and combined VF test was excellent in the scotoma lesion area. (**B**) In the paracentral scotoma group, there was excellent agreement between the combined VF test and the 10-2 VF test. (**C**) In the paracentral and nasal scotoma group, the VF tests revealed excellent agreement existed between them. The figure was prepared using the Excel and PowerPoint program in Microsoft Office 365 (https://www.office.com/, freely available for teachers at qualified academic institutions).

Point-wise correlations between the combined SAP and 24-2 SAP or 10-2 SAP using the ICCs were mostly moderate to excellent (Fig. 4). In the peripheral nasal step group, the agreement was excellent in the location of the inferior nasal scotoma between the combined SAP and 24-2 SAP. The SAP 10-2 showed excellent consistency with the combined SAP at some superior paracentral test points in the peripheral nasal step group. In the paracentral scotoma group, each point on SAP 10-2 revealed excellent agreement overall with those on the combined SAP. Finally, in the paracentral and nasal scotoma group, the agreements between the combined SAP and 24-2 SAP or 10-2 SAP were generally excellent.

## Discussion

In this study, 24-2 and 10-2 VF tests simultaneously performed in combination demonstrated a superior topographic structure–function relationship in some sectors when compared with those individually performed in glaucoma patients with isolated peripheral nasal step or paracentral and nasal scotoma.

Within the central 10-degree VF where 30% of retinal ganglion cells are posted, 24-2 VF tests allocate only 12 checkpoints^9^. There is a concern that 24-2 VF tests could be insufficient means by which to estimate glaucomatous damage to the macula. Therefore, we created a customized VF adding all points of the 10-2 VF, in which the test points are spaced every two degrees, into the central 10-degree area of the 24-2 VF test.

The combined VF test exhibited a decreased MS when compared with the 24-2 or 10-2 VF tests in the paracentral and nasal scotoma group. This might result from the fact that the combined VF represented glaucomatous VF defects more thoroughly because it included more densely spaced test points within 10 degrees and outside of 10 degrees. The combined VF test applied the full threshold strategy since the SITA program was not available for use for customized VF tests. The reduced MS of the combined VF test might result from the full threshold strategy because the full threshold strategy is time-consuming for examiners to conduct and prone to tiredness, which might be related to decreased sensitivity^10,11^.

The structure–function analysis was adopted to compare the combined VF test to the individual 24-2 or 10-2 VF test because the MD, PSD, or pattern deviation map was not provided on the customized combined VF test by the Humphrey VF analyzer. In the peripheral nasal step group, the combined VF test was superior to the 10-2 VF test and similar to the 24-2 VF test in the superotemporal sector in the topographic structure–function relationship with peripapillary RNFL thickness. In terms of the structure–function relationship, the 10-2 VF test may be insufficient to detect glaucomatous damage in glaucoma patients with peripheral nasal step. This corresponds to the Tryanis et al.’s study, which found that the 10-2 SAP can miss VF defects that are detected by the 24-2 SAP^2^. About 20% of eyes categorized as abnormal on the 24-2 SAP were normal on the 10-2 SAP and this study exemplified the situation with peripheral nasal step on 24-2 SAP^2^.

In the paracentral and nasal scotoma group, the inferotemporal and inferonasal GCIPL thickness showed better correlations with the combined SAP than with the 24-2 SAP. The favorable outcome with the combined VF test corresponds to Ehrlich et al.’s study^4^, where it was reported that adding four or 16 points (even or empirical) from the 10-2 (two-degree spacing) to the 24-2 VF test pattern led to better discrimination ability for glaucoma detection than that seen with the conventional 24-2 VF test^4^. This study used pre-existing 24-2 and 10-2 VF tests, retrospectively added some points from the 10-2 VF test to the 24-2 VF test, and did not reveal the information about the glaucoma stage or the VF defect type. We compared the simultaneously performed combination VF test with the individually performed 24-2 or 10-2 VF tests and included the subjects with early to late-stage glaucoma and various types of VF defects.

Point-wise correlations between individual 24-2 or 10-2 VF tests and the combined VF ranged generally from moderate to excellent yet were especially excellent in the paracentral and nasal scotoma group. Point-wise correlation between individual 24-2 or 10-2 VF and the combined VF demonstrated better agreement regarding the scotoma location. For the nasal step group, excellent consistency was presented in the site of nasal scotoma during the 24-2 VF test. For the paracentral scotoma group, the 10-2 VF test displayed excellent agreement in the central 10-degree area in conjunction with the combined VF test.

The size and location of VF stimulus are the same between with the combined SAP and the individual 24-2 or 10-2 SAP, respectively. A test–retest variability using SAP has been known to increase with reductions in sensitivity^12^. When the test points are near or lower than 0 dB, however, the variability decreases somewhat^12^. At the location of the scotoma lesion on the PSD of 24-2 SAP, the VF sensitivity on the threshold printout might be near 0 dB. The neighboring test points outside the scotoma on the pattern standard deviation of the 24-2 VF test might experience glaucomatous damage. Therefore, point-wise correlations between individual 24-2 or 10-2 VF tests and the combined VF seemed to be excellent, especially in the area of scotoma on 24-2 VF tests, and generally moderate in other areas besides the scotoma lesion. However, this is only our speculation further study is needed to confirm that.

One of the limitations of this study is that the combined SAP was performed using the full threshold strategy since the SITA program was unavailable for customized VF tests. It has been found that the MS is lower when using the full threshold strategy in comparison with using the SITA standard strategy^13,14^. Sharma et al. found that the SITA and full threshold strategies are comparable to one another in detecting glaucomatous damage, even though it is controversial^13–15^. The necessary test-taking time for the combined VF test was longer than the individual 24-2 or 10-2 VF test-taking times both due to increased test points and the use of the full threshold strategy. This might lead to greater fatigue effects with the combined VF than the individual 24-2 or 10-2 VFs. However, the topographic structure–function relationship between RNFL or GCIPL thickness with VF tests was not inferior to the combined VF test.

In summary, the concurrently performed combined VF test was better considering some topographic structure–function relationship parameters when compared with the individual 24-2 or 10-2 VF tests, even though it required more test-taking time. However, the superior topographic structure–function correlations of the combined VF test at some sectors with structural measurements on glaucoma did not imply that it was the supreme perimetry approach for detecting glaucomatous damage. At least, our results seem to indicate that the combined 24-2 and 10-2 perimetry with more test points might be not inferior to any individual VF test with regard to the structure–function relationship in glaucoma patients with different types of scotoma.

## References

[1] Alward WL (2000). Frequency doubling technology perimetry for the detection of glaucomatous visual field loss. Am. J. Ophthalmol..

[2] Traynis I (2014). Prevalence and nature of early glaucomatous defects in the central 10 degrees of the visual field. JAMA Ophthalmol..

[3] De Moraes CG (2017). 24-2 visual fields miss central defects shown on 10-2 tests in glaucoma suspects, ocular hypertensives, and early glaucoma. Ophthalmology.

[4] Ehrlich AC, Raza AS, Ritch R, Hood DC (2014). Modifying the conventional visual field test pattern to improve the detection of early glaucomatous defects in the central 10 degrees. Transl. Vis. Sci. Technol..

[5] Phu J, Kalloniatis M (2020). Ability of 24-2C and 24-2 grids to identify central visual field defects and structure-function concordance in glaucoma and suspects. Am. J. Ophthalmol..

[6] Leite MT (2012). Structure-function relationships using the Cirrus spectral domain optical coherence tomograph and standard automated perimetry. J. Glaucoma..

[7] Garway-Heath DF, Holder GE, Fitzke FW, Hitchings RA (2002). Relationship between electrophysiological, psychophysical, and anatomical measurements in glaucoma. Invest. Ophthalmol. Vis. Sci..

[8] Fleiss JL (1986). The design and Analysis of Clinical Experiments.

[9] Park SC (2013). Parafoveal scotoma progression in glaucoma: humphrey 10-2 versus 24-2 visual field analysis. Ophthalmology.

[10] Hudson C, Wild JM, O'Neill EC (1994). Fatigue effects during a single session of automated static threshold perimetry. Invest. Ophthalmol. Vis. Sci..

[11] Searle AE, Wild JM, Shaw DE, O'Neill EC (1991). Time-related variation in normal automated static perimetry. Ophthalmology.

[12] Wall M, Woodward KR, Doyle CK, Artes PH (2009). Repeatability of automated perimetry: a comparison between standard automated perimetry with stimulus size III and V, matrix, and motion perimetry. Invest. Ophthalmol. Vis. Sci..

[13] Bengtsson B, Heijl A (1999). Comparing significance and magnitude of glaucomatous visual field defects using the SITA and full threshold strategies. Acta Ophthalmol. Scand..

[14] Sharma AK, Goldberg I, Graham SL, Mohsin M (2000). Comparison of the Humphrey swedish interactive thresholding algorithm (SITA) and full threshold strategies. J. Glaucoma.

[15] Budenz DL (2002). Comparison of glaucomatous visual field defects using standard full threshold and Swedish interactive threshold algorithms. Arch Ophthalmol..

